# Building trust and relationship across language barriers: a qualitative study of interpreter-mediated psychotherapy with therapists, interpreters and patients

**DOI:** 10.1186/s12939-025-02718-6

**Published:** 2025-12-05

**Authors:** Muhammed-Talha Topçu, Mike Mösko

**Affiliations:** 1 Department of Applied Human Sciences, University of Applied Sciences Magdeburg- Stendal, Osterburger Straße 25, 39576 Stendal, Germany; 2 Department of Medical Psychology, University Medical Center Hamburg-Eppendorf, Hamburg, Germany

**Keywords:** Interpreter-mediated psychotherapy, Language barriers, Mental healthcare, Therapeutic alliance, Trust

## Abstract

**Background:**

Among a variety of barriers to accessing and providing mental health care for migrant and refugee populations, language barriers play a crucial role. Interpreter-mediated psychotherapy (IMP) has emerged as an effective approach to overcoming these challenges and promoting equitable access to mental health care. However, IMP also introduces a more complex relationship structure: the classical therapist–patient dyad evolves into a triad. Relational qualities such as trust, empathy, and mutual understanding are known to be key indicators of a positive therapeutic relationship. Despite the utilisation of interpreters, research on the relational qualities of IMP remains limited. While several qualitative studies have explored relational dynamics in IMP, few have examined these from the perspective of all parties, particularly by incorporating patient’s perspectives. Therefore, this study explores the relational qualities in IMP, focusing on trust-building, the role of nonverbal communication, and relationship development among all parties involved, in order to inform practices that foster equity in mental health care for patients facing language barriers.

**Methods:**

To address this, 21 semi-structured interviews were conducted with mental health care providers (*n* = 6), interpreters (*n* = 6), Turkish- and Arabic-speaking patients (*n* = 6), and three experts in migration and mental health, relationship building in psychotherapy, and translation studies. The interviews were audio-recorded, transcribed verbatim, and analysed using qualitative content analysis.

**Results:**

12 relational qualities emerged from analyses of the triadic relationship. Across all relationships, trust emerged as a central theme for all participants. Within the patient-therapist dyad, clear information about confidentiality fosters patient openness, while patients particularly value nonverbal communication alongside the therapist’s emotional presence and responsiveness. In the interpreters’ relationship with both therapist and patient, the importance of accurate translations and adherence to confidentiality appeared to be the most influential factor in building trust among the involved parties.

**Conclusion:**

This study offers insights into how relational qualities, such as trust, operate across different relationships within IMP. It highlights the role of IMP in advancing equitable mental health care and demonstrates that, despite potential uncertainties, therapeutic relationships and trust can be effectively established when language barriers exist. These insights offer concrete starting points for developing guidelines and trainings both for practitioners and interpreters, aimed at fostering inclusion, mutual understanding, and improving the overall quality of mental health service delivery in settings with language barriers.

**Clinical trial number:**

Not applicable.

## Overcoming language barriers

Language is the therapeutic tool in mental health care and a central component of patient healing [1] as it enables mutual understanding and is crucial for relieving patients [2], making a shared language between therapist and patient indispensable for successful treatment [3]. Against this background, and in light of increasing globalisation, migration and global crises, the issue of psychosocial interventions for refugees and other migrant populations and their access to (mental) health care in host countries, has come to the forefront of global mental health research. According to current figures, the number of refugees worldwide has reached a record high [4]. Despite the need of mental health treatments, refugees and migrant populations remain significantly underrepresented in the utilisation of such services [5]. Research highlights various barriers to access mental health treatments, with language barriers playing a key role [6, 7].

In order to overcome language barriers, therapy that includes bilingual, or multilingual treatment options would likely be the most desirable solution. Although studies show that bilingual or multilingual mental health care providers already tend to treat multilingual patients more frequently than average [8], there is a significant shortage of bilingual therapists. As a result, many patients cannot receive treatment in their native language [9]. Language barriers are therefore one of the most significant obstacles to accessing mental health care for migrants and refugees. Possible consequences of this challenge include misdiagnoses, legal and bureaucratic hurdles, and cultural discrepancies between therapists and patients with a migration background [9]. Additionally, language barriers can negatively impact treatment outcomes and lead to difficulties in fostering empathy [10]. Besides employing multilingual professionals, another way to overcome language barriers is through the use of technological or digital tools [11], although they come with significant challenges, such as the accurate translation of medical terminology [12]. Additionally, to overcome communication barriers and the associated challenges, people are often forced to rely on family members, friends, or acquaintances or multilingual professionals working in clinics who act as lay interpreters [13, 14]. However, the use of lay interpreters has proven to be problematic due to challenges such as the inaccurate translations and concerns regarding confidentiality and ethics [14, 15]. For these reasons, interpreter-mediated psychotherapy (IMP) should be employed through professional and qualified interpreters.

### Interpreter-mediated psychotherapy (IMP)

Interpreters provide access to the emotional world of patients, which would not be possible without their support [16]. The use of interpreters also allows patients to communicate in their own language, thus reducing misunderstandings [17]. From the patients’ perspective, interpreting is a useful support to convey information and to make oneself understood [13]. Especially with regard to culture-specific content, interpreters can act not only as linguistic bridges but also as cultural mediators [18]. Despite the lack of research in this field, studies suggest that the use of (professional) interpreters improves therapy [19, 20]. Lambert and Alhasson [21] examined the results of randomized controlled studies on psychotherapeutic interventions for traumatized adult refugees in their meta-analysis and found no differences between studies with and without an interpreter present. Another study by Brune et al. [22] reached the same conclusion, that interpreter-mediated psychotherapy sessions are at least as effective as monolingual therapies. However, a more recent study could not confirm these positive effects and attributes them to difficulties in the therapeutic relationship [23]. This particularly is one of the concerns associated with interpreter-mediated psychotherapy. Therapists are often hesitant to employ interpreters [24], a reluctance that can stem from structural challenges, such as lack of funding and insufficient training [25, 26] or from subjectively perceived concerns related to the establishment of the therapeutic relationship and the complex dynamics in interpreter-mediated psychotherapy [16, 17].

### From the dyadic relationship to the triad

The therapeutic relationship is considered one of the central, active factors, contributing consistently and significantly to positive treatment outcome [27, 28]. This relationship, often referred to as alliance, which originates from the psychodynamic tradition and was defined by Bordin [29] as a working alliance, consisting of three core components: an emotional bond between patient and therapist, agreement on therapy goals, and consensus regarding the therapeutic tasks. Within IMP settings, this relationship between therapist and patient becomes a triad between therapist, interpreter, and patient. The dyadic relationship now turns into a much more complex relational web, characterised by mutual sub-dyads between (i) therapist-patient, (ii) therapist-interpreter, (iii) interpreter-patient, and (iv) the therapist-patient-interpreter triangle [30, 31]. While in a dyadic configuration, the responsibility for relationship-building was often attributed to the therapist, in the triad, the relationship is also influenced and shaped by the interpreter [32]. This triadic configuration of therapist, interpreter, and patient is associated with both specific challenges and significant opportunities [1]. In addition to the aforementioned opportunities in interpreter-mediated psychotherapy, specific challenges have been identified, particularly concerning the relationship-building process, where therapists may feel excluded [30] and may fear losing control due to a too-close relationship between the patient and the interpreter [10]. Interpreters, on the other hand, sometimes perceive the relationship with therapists as disrespectful, culturally insensitive, and feel that their role is misunderstood [33, 34].

### Trust in IMP

Trust and empathy are considered essential factors for a successful therapeutic relationship [35]. In nearly all studies dealing with relationship issues in interpreter-mediated psychotherapy, trust is seen as a key component [16, 30, 36–42]. At the same time, a lack of trust on the part of patients towards both the therapist and the interpreter can lead to the concealment of relevant information for the session or even result in the patient not seeking treatment at all [16, 43].

In regard to empathy, there are concerns that the emotional involvement might overwhelm the interpreter [37]. Equally, a good relationship between the interpreter and the patient is considered necessary for transferring the relationship to the therapist [10, 43]. For therapists, nonverbal communication – expressed through nodding, eye contact, and facial expressions – becomes increasingly important in this setting to establish a strong therapeutic relationship with patients [30]. In their systematic review, Geiling et al. [43] further conclude that building trust requires time and that, besides body language, the cultural and linguistic closeness of the interpreter to the patient is particularly trust-enhancing. Also, Delizée and Michaux [44] investigated in their research how the (therapeutic) relationship is constructed in IMP and conclude that the therapist-interpreter relationship is characterised by mutual trust and support, the interpreter-patient bond consists of trust, security and attachment and that the therapist-patient dyad is influenced by the other two dyads, but also consists of trust and security.

Given the limited research on these aspects, it remains unclear how trust, empathy, and other indicators of a strong therapeutic relationship are built, threatened, and recognised in IMP, and how the members of the triad perceive and describe their relationships within the sub-dyads. Therefore, the goal of this study is (i) to gain insight into the perception of the respective relationship, taking important relationship qualities into account, and (ii) to explore how central relationship qualities such as trust are conveyed, negotiated, and perceived by the different participants of the triad.

## Materials and methods

### Participants

To investigate the research question, this study employed a qualitative interview study with a semi-structured interview guide. A key objective of this research was to gather insights from all participants within this triadic relationship, providing a comprehensive perspective on the topic. Consequently, this study included mental health professionals, interpreters, and patients. Mental health professionals must have (a) worked in the field of psychotherapeutic or psychosocial care, (b) hold a university degree in medicine, social work, or psychology and (c) either be registered as psychological or medical psychotherapists or psychiatrists, (d) currently undertaking training or specialisation in one of these areas. Furthermore, they must have experience in interpreter-assisted psychotherapy, with a minimum of three completed sessions. This study exclusively included professional or formal interpreters. Given that interpreting is not a regulated profession with standardised qualifications or a clearly defined legal framework, we defined professional interpreters as those for whom interpreting in healthcare is the primary occupation, with at least two years of experience and experience in at least three IMP sessions. Family members, friends, and other lay interpreters were thereby excluded. Regarding the patient group, Turkish- and Arabic-speaking patients were recruited who were either currently or previously in psychotherapeutic care with the support of an interpreter. The selection of these languages ensured the inclusion of the largest migrant populations from Turkey and the largest refugee population from Syria in Germany and enabled our research team to conduct interviews in the patients’ native languages. This approach aimed to facilitate effective communication and capture nuanced perspectives. Additionally, to these groups, three experts were interviewed, one for each subgroup. These experts included distinguished scholars specializing in therapeutic relationships, representing the perspective of mental health professionals; scholars focusing on interpreting within psychotherapy, representing the interpreters’ perspective; and professionals with extensive experience in providing psychotherapeutic care to migrants, representing the perspective of migrant patients.

### Recruitment

An overarching attempt to recruit therapists, interpreters and patients was to send invitations to participate in the study, along with study information sheets, via email directly to therapists in North Germany, psychosocial centres, who offer interpreting services and interpreter agencies providing services within the healthcare sector. A combination of purposive sampling and snowball sampling was employed to enhance the likelihood of reaching suitable participants. Additionally, a wide range of strategies was employed to reach patients. Study information sheets were distributed in community settings, including language schools, mosques, and restaurants, to implement a community-based approach. All participants reached the research team via email or phone calls and declared interest in the study. Prior to interviews, all participants received oral and written information about the study in either German or Arabic or Turkish. All participants provided written, informed consent for their interviews to be audio recorded, transcribed, and analysed for research purposes. This study did undergo ethical approval at University of Applied Sciences Magdeburg-Stendal.

### Interview guide

The interview guide was developed following a literature review by MTT, in consultation with and under the supervision of MM. A general interview guide was created covering broad topics such as role understanding, experiences in interpreter-mediated psychotherapy, perceptions of the therapeutic relationship, and expectations of the triad. The questions were adapted for each group to ensure they were appropriate to their specific perspective. Open-ended questions were used for this purpose. One section of the questions focused on describing the relationship with the other two parties, aiming to gain a more detailed and in-depth understanding of relationship qualities within interpreter-mediated settings. For example, we asked the interpreters to describe a good relationship with patients and a good relationship with therapists (e.g., *How would you describe a good relationship between you and the patients in this setting?*). Accordingly, these questions were adjusted for therapists and patients. Another objective was to better understand how relationship-building (e.g., *How do you establish a good therapeutic relationship with your patients?*) and the communication of aspects such as trust (e.g., *How do you recognise that a patient trusts you?*) are facilitated.

### Data collection

The interviews were conducted between April and November 2024. The interviews took mainly place in-person. Two interviews were conducted online via zoom as participants were not able to make it in person. The patient interviews took partly place in places which were convenient to the patients, e.g. at their homes. All interviews were conducted in German, except the patient interviews. One of our research staff, who is fluent in Arabic, conducted the interviews with the Arabic-speaking patients in Arabic, and the first author conducted one interview in Turkish with the Turkish-speaking patient, which gave us the opportunity to directly communicate with this group without recreating the setting of an interpreter-mediated conversation. The duration of the interviews ranged from 23 min (minimum) to 62 min (maximum), with an average length of 48 min. The interviews were transcribed with an audio-recorder and transcribed verbatim [45] with the help of trained student assistants. Translations of the patient’s interviews from Turkish and Arabic into German were done by professional translation offices and cross-checked by the research team.

### Data analysis

The transcribed interviews were analysed following Braun and Clarke’s [46] thematic analysis approach using MAXQDA 2024. Themes were generated inductively based on the interview data in relation to the research questions. To ensure methodological rigor and adherence to quality standards in qualitative research, the interviews were independently coded by MTT and MAK, a research intern specifically trained for this task. Both coders initially familiarized themselves with the data by reading the transcripts, generating initial codes, and subsequently developing broader themes. After independently coding six interviews, the two coding systems were compared, discussed, and consolidated into a unified framework. To ensure inter-coder reliability, coding was cross-checked, and any discrepancies were resolved through consensus in biweekly team meetings. Researchers’ reflexivity regarding cultural and linguistic positions was also addressed during these meetings. Additionally, codes were discussed within the larger research group to further enhance reliability. The remaining interviews were then analysed by MTT, under the ongoing supervision of MM.

## Results

At the beginning, we discussed the complexity of the triadic relationship structure, which also presents a challenge when it comes to presenting the results. To ensure clarity in conveying the findings derived from this complex data because of the different angles of it, we have chosen the following approach: We have decided on a content-oriented presentation of the results rather than an actor-oriented one. This means that we will first present themes that were relevant to all three perspectives—therapist, interpreter, and patient—and were mentioned in the interviews from these perspectives. Additionally, under each overarching theme, the perspectives of the respective actors will be highlighted when necessary to illustrate nuances and differences in relation to the theme. Afterwards, we will present themes that were particularly relevant to specific group of actors. The evaluations of the interviewed expert will be incorporated into the discussion, where their perspective is considered valuable. This allows us to focus on the needs and experiences of each group. With this approach, we aim to make the presentation of results for this complex relational structure more comprehensible.

### Sample

The study included four subgroups: interpreters (*n* = 6), mental health professionals (*n* = 6), patients (*n* = 6) and experts (*n* = 3). Six interpreters participated, with ages ranging from 34 to 53 years (*M* = 44 years). The group included five females and one male. The interpreters spoke a variety of languages, including Turkish, Romanian, Russian, Galgaya, Chechen, Farsi, and Dari. Their professional experience ranged from 3 to 28 years. The mental health professionals represented a diverse range of experience levels, educational backgrounds, and areas of specialisation. Among them, there is one psychiatrist (*n* = 1), two behavioural psychotherapists (*n* = 2), and one psychodynamic psychotherapist (*n* = 1). Additionally, two of the participants are psychotherapists currently in training, both specialising in behavioural therapy (*n* = 2). A total of six patients participated, with ages ranging from 34 to 53 years (*M* = 44 years). The patient group consisted of five males and one female. Most service users spoke Arabic, with one service user speaking Turkish. Additionally, experts in migration and mental health (*n* = 1), relationship building in psychotherapy (*n* = 1), and translation studies (*n* = 1) were also interviewed.

### Overview of relational qualities in Interpreter-mediated psychotherapy (IMP)

**Fig. 1:**
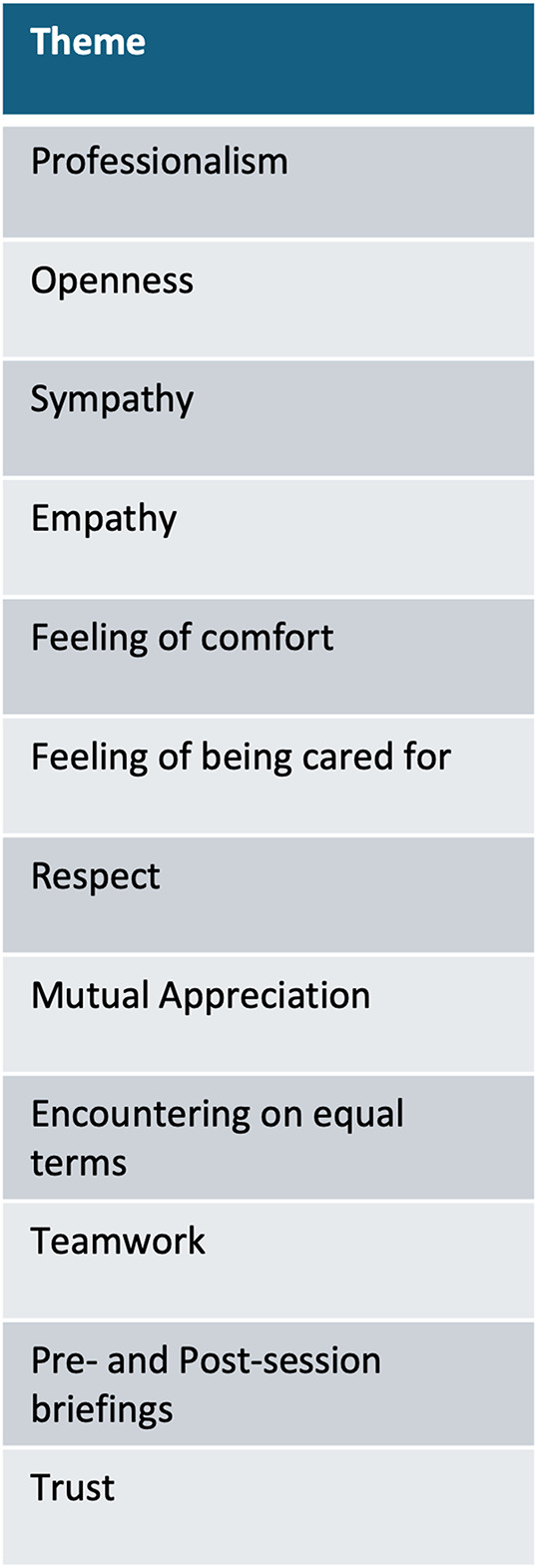
Identified overarching themes regarding relational qualities in IMP

Under the main goal of examining the relational qualities, 12 themes of relational qualities have been developed, under which several subthemes have been found. One of these themes concentrates on the aspect of trust, with 3 subthemes and several sub-subthemes on (I) how to build trust, (II) indicators of trust-based relationships, and (III) difficulties and barriers in building trust. Throughout the interviews several other themes, such as (a) difficulties in IMP, (b) comparisons to monolingual therapy, (c) role perceptions and descriptions or (d) role conflicts were identified but will not be part of this analysis as this is going beyond the scope of this paper. An overview of the identified relational qualities can be found in Fig. 1.

#### Professionalism

A commonly shared theme across all participants was professionalism. While patients associate professionalism primarily in their relationship with the interpreter, they state a good relationship with the interpreter is a professional one. In this context, professionalism is also linked to the conscientiousness of the interpreter’s translation work. A fundamental requirement for interpreters to provide accurate and diligent translations is that they display professionalism. While the practice of the profession of the interpreters is described by the patients through professionalism, interpersonal relationships are often described as friendly and familial (“like a brother/sister”). From the therapists’ perspective, professionalism relates more to their own therapeutic attitude, which is characterised, among other things, by maintaining a certain distance while still fostering closeness in the relationship with the patient. Only in this way can patients feel safe enough to open up: *“Some people say*,* ‘Oh*,* yes*,* the therapist is like my best friend.’ I wouldn’t see it that way. It’s about maintaining professionalism so that the patient can say things they wouldn’t say to their best friend.”* (Therapist 2).

Therapists also emphasised that they see themselves as being closer to the patient than to the interpreter, where the relationship primarily maintains a professional character. Interpreters, on the other hand, related the aspect of professionalism to the therapeutic attitude, with a particular emphasis on impartiality. Professionalism was further characterised by punctuality, a neat appearance, and the use of formal address.

#### Openness, sympathy, and empathy

Among the overarching (global) themes identified in relation to interpersonal dynamics in IMP, openness, sympathy, and empathy emerged consistently across participants. While patients described openness as a fundamental prerequisite for meaningful exchange within the therapeutic triad, therapists predominantly framed openness as a tool for establishing rapport. Through open-ended questioning and a genuine sense of curiosity, therapists can foster a connection with patients. This openness may pertain both to the patient’s personal narrative and to cultural aspects that may be unfamiliar to the therapist. Interpreters likewise emphasized openness as a key relational quality. However, in their accounts, openness primarily referred to the therapist–interpreter relationship and encompassed a willingness to communicate transparently about uncertainties or ambiguities, thereby promoting collaborative dialogue.

Empathy was also identified by both therapists and interpreters as a crucial quality for effective collaboration in the triad. Participants stressed the importance of maintaining professionalism while simultaneously extending a sense of empathy toward one another.

By contrast, sympathy was primarily mentioned by interpreters and therapists as a relational quality essential for their successful collaboration. It was associated with interpersonal “*chemistry*” and mutual appreciation, which were seen as important for facilitating a cooperative working atmosphere.

#### Feeling of comfort and being cared for

Another theme that was mentioned by all parties involved, but particularly by almost all of the patients, was the aspect of comfort. This sense of comfort and security relates not only to the relationship with the therapist but also to the relationship with the interpreter. Both interpreters and therapists have stated that the focus should be on ensuring the patient feels comfortable, and that their well-being should be prioritised.

As a result, patients often reflect that they feel relieved and at ease: *“I often told her [the therapist]*,* ‘When I see you and the interpreter*,* I feel comfortable because I can talk and get everything out of me’*” (Patient 2). At the same time, the opposite is also seen as a reason for the termination of therapy: *“(…) if the patient does not feel comfortable with the therapist*,* they stop attending. They no longer come back.”* (Patient 5).

#### Respect, mutual appreciation, and encountering on equal terms

Respect for the respective participants in the triadic setting has emerged as another key aspect of the relationship. Respect here broadly refers to the person themselves, as well as what the person brings with them, such as cultural differences. According to the therapists, it is important to adopt a culturally sensitive approach and respect these differences. Respect also plays an important role between the therapist and the interpreter, which is reflected in small acts, such as *“offering the chair when there is no chair for the interpreter”* (Interpreter 6), and goes hand in hand with the appreciation of the other person. This respect and appreciation, in interpreter-therapist relationships, also extends to valuing the work of the other.

Patients further report that therapeutic care contributes to building a strong relationship with the therapist. This is expressed in the way therapists care for the patient as a person, making them feel important, showing concern for them, and asking about the patient’s everyday situations and family matters without being judgmental: “*She gives me the feeling that I am an important person to her.”* (Patient 1). Patients also mentioned that interpreters should also *care* for the patients.

For patients, this is particularly evident when therapists and interpreters meet them on *equal terms*. This approach, characterised by gestures such as the therapist apologising for cancelled appointments or personally picking the patients up from the waiting room before sessions, leads to patients feeling valued and developing an intimate connection with the therapist, which is ultimately conducive to building trust: *“I felt that she was hurt because the appointment had to be cancelled because the interpreter didn’t show up. And this situation really increased my respect for her. She was upset and regretted it. Sometimes she apologises a lot*,* and that’s something I didn’t expect.”* (Patient 2). Meeting on equal terms is also regarded as particularly important for the therapist-interpreter relationship: *“So*,* on equal terms. In general*,* being on equal terms is crucial. It’s important not only in relation to the patient but also in relation to the therapist or doctor. I am the expert in my field*,* and my counterpart*,* the therapist*,* is the expert in their field*,* and for me*,* the patient is the expert in their own life. I think this equality is the foundation.”* (Interpreter 2).

#### Teamwork

These themes were particularly relevant to the interpreter-therapist relationship, where both therapists and interpreters largely agreed on the importance of perceiving the work in (IMP) as a collaborative effort. This approach entails cooperation and a clear understanding of roles within the triad. While there appeared to be a shared understanding of these aspects between therapists and interpreters, nuances emerged between their perspectives.

Interpreters frequently reported the challenge of balancing the differing expectations of both therapists and patients. As a result, there was a strong desire for clear communication before therapy sessions to ensure that each member of the triad fully understands their role in the setting. This clarity of roles goes beyond simply defining individual responsibilities; it also encompasses (a) an understanding of each other’s working methods and (b) alignment on the common goals for the sessions, which should ideally be discussed and clarified during pre-session meetings between the interpreter and therapist.

#### Pre- and post-session briefings

This aspect was primarily highlighted by interpreters and therapists and predominantly pertains to the interpreter-therapist relationship. It encompasses elements such as mutual awareness of burdens, needs, and expectations, which are either acknowledged or actively addressed by the other party. One central approach to demonstrating awareness of the interpreter’s needs, particularly in recognizing the emotional toll of the sessions, is the implementation of pre- and post-briefings. Pre-session meetings are particularly useful for establishing rapport with the interpreter and discussing any specific needs or concerns they might have. Meanwhile, post-session briefings serve as an opportunity to address the interpreter’s mental well-being, offering space for reflection and emotional processing. However, beyond inquiring about the interpreter’s well-being, therapists reported that inviting interpreters to share any relevant observations or concerns that emerged during the session to be equally important in post-session briefings. Although nearly all interpreters expressed a desire for pre- and post-briefings and even reported instances where they proactively requested such meetings, the responsibility for initiating them primarily falls on the therapist. When therapists take this initiative, interpreters feel acknowledged and valued, which in turn positively influences the interpreter-therapist relationship: *“For example*,* when the therapist asks after the therapy session*,* ‘So*,* how are you? How did you cope with that?’ or something along those lines. It doesn’t have to be much*,* but it helps the interpreter know*,* ‘Aha*,* the therapist is there for me in case I struggle to set boundaries.’“* (Interpreter 3).

### Trust

**Fig. 2:**
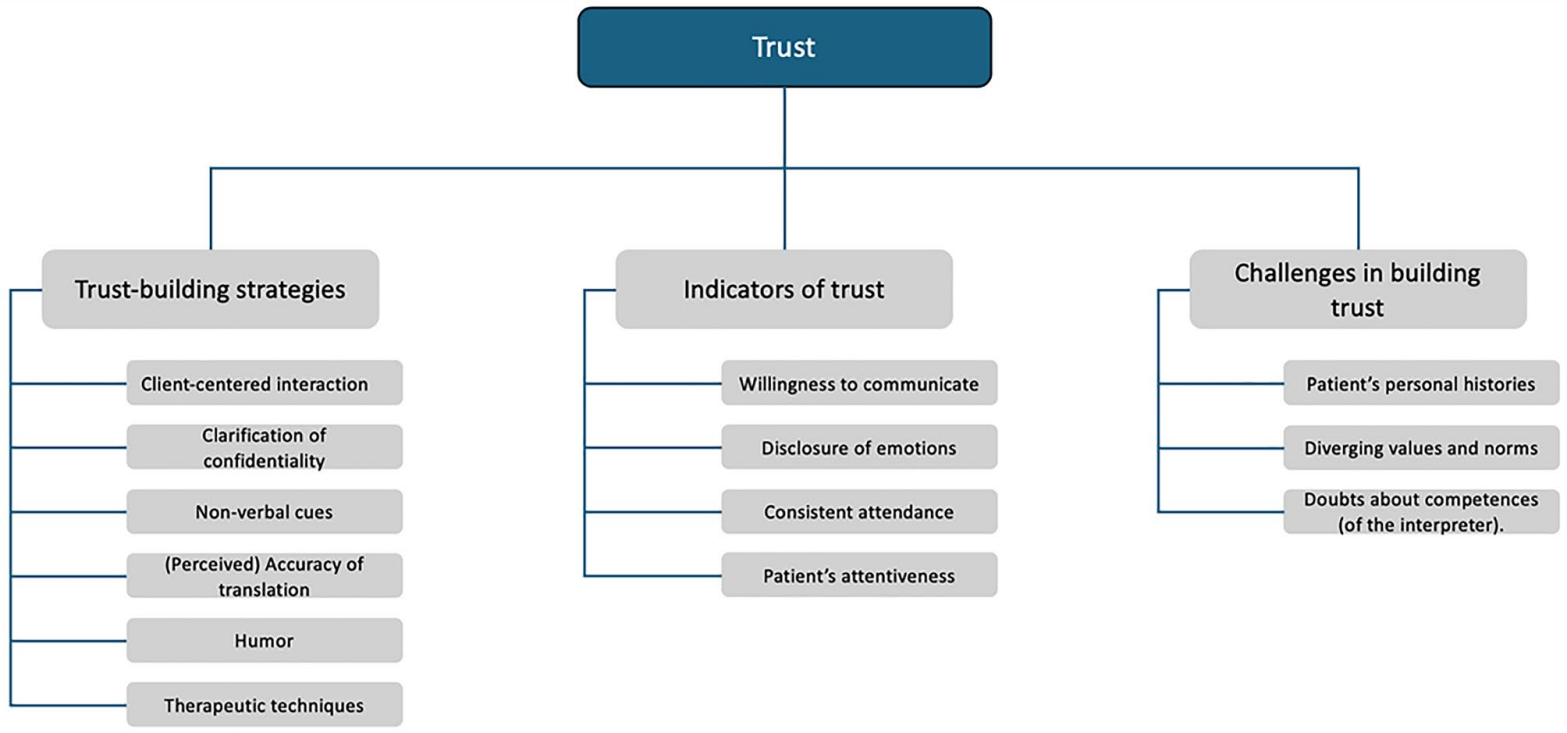
Identified subthemes and sub-subthemes under the overarching theme of trust

Almost all participants identified trust as an important relational quality within the triadic relationship. However, the function of trust appears to vary depending on the specific dyadic relationships within this structure. For instance, therapists often emphasize the necessity of trust in the therapist-patient relationship, as it may facilitate patients in opening up about their needs and mental health concerns. In contrast, trust in the therapist-interpreter relationship tends to be associated more with the interpreter’s professionalism and accuracy of translations. This suggests that trust may function as an umbrella term encompassing distinct expectations and roles shaped by the specific needs of each dyadic relationship. Identified subthemes and sub-subthemes under the overarching themes of trust can be found in Fig. 2.

Notably, these different functions of trust seem to be also commonly shared across the different parts of the triadic relationship. For example, some interpreters also describe trust in the therapist-interpreter relationship as being rooted in professional competence: *“I think the therapist has to trust the interpreter 100% that exactly what he says will be interpreted*,* that he won’t add anything*,* that he won’t leave anything out (…)” (Interpreter 1)*.

Similarly, the therapists’ perspective is also reflected in patients’ accounts, as they describe trust as a prerequisite for discussing their personal experiences with the therapist: *“Because of the trust between us (…) I open my heart and tell her everything I want.”* (Patient 2).

#### How to build trust?

***Patient-centered interaction***. Showing interest in the patient or interpreter as an individual was described as a way for therapists to build trust. By asking questions about personal circumstances before initiating diagnostic or therapeutic procedures or by learning a few words in the patient’s language, therapists reported making efforts to establish trust. This approach was also reflected in patients’ interviews and mentioned as trust-building. Likewise, to create a good atmosphere that encourages trust and openness without imposing any pressure was reported to be of importance. In addition to therapists’ efforts to ease into the conversation gradually, spatial factors—such as open spaces, offering something to drink, and similar gestures—also contribute to fostering this sense of comfort.

***Clarification of confidentiality obligations.*** An effective factor that was equally perceived as beneficial by all actors was the clarification of confidentiality obligations prior to the therapies. By informing participants about the confidentiality of both therapists and interpreters and assuring them that no information would be shared with unauthorized third parties, a secure space for patients can be created: *„Those of us who live by the sea say: we sit by the sea and talk to the sea. Why? Because there will be no reaction and no problems afterwards (…) I think that the therapist is like the sea for me because I can be certain that the information I will share with them will be absolutely safe and confidential. This means that there is nothing to worry about.“ (Patient 1).*

This clarification of confidentiality not only encouraged patients to discuss topics they had previously withheld due to concerns but also facilitated open communication with both the therapist and interpreter. Conversely, a breach of confidentiality or even doubt about an participants discretion appeared to hinder the establishment of trust from the outset.

***Non-verbal cues***. Another crucial aspect shared by all participants is the role of nonverbal communication. Through body language—such as an *open posture*, *facial expressions*, and *gestures* when explaining certain things to patients—not only do patients gain a better understanding of the therapist and interpret their reactions more accurately, but they also receive a signal of being understood, which patients consider particularly important: *„[The interpreter] translates the information and I can easily tell how affected the therapist is when he conveys information to her. Then I have the feeling that she has been given the right information.“* (Patient 2). This includes emotional reactions for therapists such as *“showing a sad face when the patient explains something sad”* (Therapist 2), which are recognized and observed as such from patients: *“And [from] their face—how they reacted with compassion.”* (Patient 2).

Additionally, the therapist’s nonverbal and emotional responses play a significant role in the interpreter-patient relationship. Patients rely on these cues to evaluate whether they can trust the translation and, by extension, the interpreter. „*One understands signs and facial expressions when people are angry*,* happy and sad by facial expressions. I have the feeling that he [the interpreter] is conveying the right information and I have the feeling that the psychiatrist understands me.*“ (Patient 2).

***Accuracy of translation***. The (subjective) perception of the participants about the accuracy of the interpreter’s translation plays a central role in the development of all relationships in this triadic structure of relationships. While a translation that is perceived as good not only establishes the therapeutic relationship with the patient, e.g. by giving patients the feeling of being understood by the therapist’s response, it also has a direct influence on the relationship between interpreter-therapist and interpreter-patient, through which trust is strengthened because both can assume that the interpreter is translating correctly and accurately: “*Through the patient’s answer and questions*,* the therapist knows anyway whether or not it has been received correctly. And vice versa too* (…). *When they know that the interpreter translates accurately*,* this is what creates trust*” (Interpreter 4).

***Humor****.* Several therapists also mentioned using humor as a way to build trust with their patients. Shared laughter helps to bridge gaps, reduce fears or the sense of authority associated with the therapist, and create a more relaxed atmosphere. Additionally, humor is said to foster a sense of connection and allows therapists and patients to engage on an *equal footing*, which was further mentioned as an important factor to build trust with both the interpreter and patient: *“On equal footing. (…) Not this top-down approach. Like*,* “I am helping you*,*” and the other person being a supplicant.“ (Interpreter 1)*.

***Therapeutic techniques***. In addition to the overarching approaches mentioned above, which were commonly perceived as useful, some therapists also highlighted specific therapeutic techniques that appear to support trust-building with patients. One such technique is *synchronization*, which involves mirroring the patient’s body language, such as their posture. This nonverbal approach was seen as a way to foster a sense of closeness and connection. *Validation* also emerged as a strategy, as it helps convey care and presence to the patient. Lastly, the *lifeline*-*technique* used in behavioral therapy training was noted as another method that facilitated trust-building. Interestingly, these specific techniques which helped the therapists to build trust to patients were only mentioned by those mental health professionals who are currently in training.

#### Indicators of trust

***Willingness to communicate and disclosure of emotions***. Regarding indicators of trust, therapists and interpreters mentioned various signs that suggest patients trust them. Almost all participants linked a trusting relationship to the patient’s willingness to communicate. This willingness, and the associated indication of trust, can be observed when patients begin to discuss topics *related to guilt and shame*, take the initiative *to bring up concerns on their own*, or actively *ask questions*: “*Everything associated with guilt and shame is often avoided at the beginning*,* including topics like violence—anything in that direction. But when they start to open up about these areas*,* even if it’s difficult for them*,* that’s when I know*,* okay*,* I’ve gained their trust.”* (Therapist 1).

This willingness to communicate extends beyond verbal expression to the disclosure of emotions. The act of emotionally opening up, such as crying but also laughing, is also regarded as a sign of trust. These were equally perceived from therapists and interpreters: *“I believe [it shows] from the events and stories that the patient shares. When it comes to very intimate and deep wounds being spoken about in the room*,* I know that such information might not be possible to share in another setting. So*,* based on the information*,* I would consider that as an indicator; otherwise*,* it’s something you can only estimate.”* (Interpreter 6).

***Consistent attendance***. Another indicator of trust was the consistency of attending therapy sessions and “coming back” to therapy, because otherwise patients would not show up: “*It is also a sign that the therapy is benefiting them. Otherwise*,* it is often observed that patients cancel or stop coming.”* (Interpreter 4).

***Patient’s attentiveness***. Another aspect is the patients’ *body language*. This includes factors such as how patients are sitting—whether, for example, they appear closed off—or whether they gesture while talking. Additionally, *eye contact* plays an important role. Therapists have described that eye contact between the patient and therapist, even when different languages are spoken, can be seen as an indicator of trust: “*So*,* she also speaks to me*,* perhaps while he or she is speaking Polish. But she looks at me and speaks to me in Polish*,* essentially”* (Therapist 3).

#### Challenges in building trust

***Patient’s personal histories.*** Several challenges were perceived as difficult when building trust within the triad. One aspect, mentioned by a patient, emphasizes the (*traumatic) life experiences* of patients, which make it generally difficult for them to establish trust: *“At the beginning*,* for someone who wanted to gain trust… The problem lay with me. The problem was that*,* for example*,* I don’t trust anyone right away. Not at all. I always keep my eyes open. Even when I sleep*,* I sleep with my eyes open (…) because life taught me not to trust anyone*” (Patient 6).

***Diverging values and norms.*** A frequently mentioned aspect was the presence of diverging values and norms, which were recognized as a barrier in all three relationships. In the interpreter-patient relationship, a *perceived mismatch in gender* between the interpreter and patient was identified as a dysfunction for building trust. This issue was attributed to differing culturally informed values. In addition to gender dynamics a mismatch in *cultural differences* or ethnic backgrounds of interpreters and patients was perceived problematic: *“(…) for example*,* because I also translate Russian/ there are many people who are coming from Ukraine now. Um*,* even though I’m not Russian or anything*,* I can speak Russian… That’s why people don’t want to stay open at first” (Interpreter 6)*.

Additionally, *divergent political views*, *perceived or assumed religious affiliations* of interpreters by patients, within the interpreter-patient relationship were also seen as factors that hindered trust-building. For instance, a Muslim patient, who assumed that the interpreter shared the same Muslim background, withheld certain information that she later shared with the therapist once the interpreter was no longer present: *“The interpreter cancelled at the last minute for a good reason. Then*,* the woman from the Muslim background said*,* ‘Yes*,* you know*,* I have a problem*,* and I couldn’t tell that to the interpreter.’ She thought the interpreter would have an issue with it because she—I don’t know whether she identifies as Muslim or not—but ultimately*,* based on her parents’ background*,* she came from a Muslim country.”* (Therapist 1).

At the same time, a seemingly appropriate match in terms of shared culture and country of origin between the interpreter and patient can also lead to difficulties in building trust. While interpreters express concerns that patients may distrust their role, thinking that *“interpreters are in cahoots with the system*,* the police*,* against the patient*,* which leads to a breach of trust” (Interpreter 1)*, a patient expressed concerns about the interpreter coming from the same cultural background, fearing that everyone would know each other, and therefore, he „(…) *would never ever speak openly about [his] problems*.“ (Patient 6).

In the therapist-interpreter relationship, especially diverging work ethics and ways of how to approach this professional working relationship was perceived as a central barrier to build trust. Also, one interpreter mentioned that discriminatory sentences from therapists or not enough cultural sensibility towards patients leads to mistrust towards the therapist.

***Doubts about competences (of the interpreter).*** Another theme that emerged across the different perspectives was the issue of doubt and mistrust, but which predominantly was mentioned regarding the qualities of the interpreter, as perceived by patients and therapists. In this context, patients particularly expressed concerns about the adherence to confidentiality, which led one patient to prefer being treated in another foreign language rather than in their mother tongue:

Unfortunately, not all interpreters are professionally honest. Not all interpreters. I’m not exaggerating. Most interpreters go to their friends after work. ‘What happened today?’ ‘This and this and this.’ There is no professional honesty at all. (Patient 6)

Maybe this person sees him, or maybe this person knows him and has seen him somewhere. I feel like he’s telling him something about his private life, and he’s sharing it with his friends or something. (Patient 5)

Both patients, especially those with some knowledge of German, and therapists expressed scepticism about the quality and accuracy of the interpreters’ translations, which led to a breach of trust:

I can speak a little German. When I feel that he is saying exactly what I have said, I feel calm and relieved. But when I sense that the words are changing, I feel irritated and suffocated. (Patient 1)

When conversations are held in the foreign language that have nothing to do with my question, and then they say ‘Yes, we discussed it,’ and I ask, ‘Yes, and now what?’ (Therapist 3)

At the same time, interpreters also perceived the scepticism regarding their ability to translate accurately, as noted by therapists, particularly when it was accompanied by follow-up questions and attempts to ensure correctness. This was viewed as detrimental to the development of trust between the therapist and the interpreter: *“As I said before*,* if the therapist doesn’t trust the interpreter*,* that’s challenging (…) and then a sense of competition arises. ‘Did you tell them this again? Did you say that?’ (…) I said*,* ‘Yes*,* I told them.’ [she responded: ]’But I didn’t hear that.’ [I said: ]’And can you say the numbers in Persian?’ ‘No*,* I can’t*,*’ she said. ‘Well.’ Hmm. So*,* such conversations have occurred.” (Interpreter 4)*.

## Discussion

**Fig. 3:**
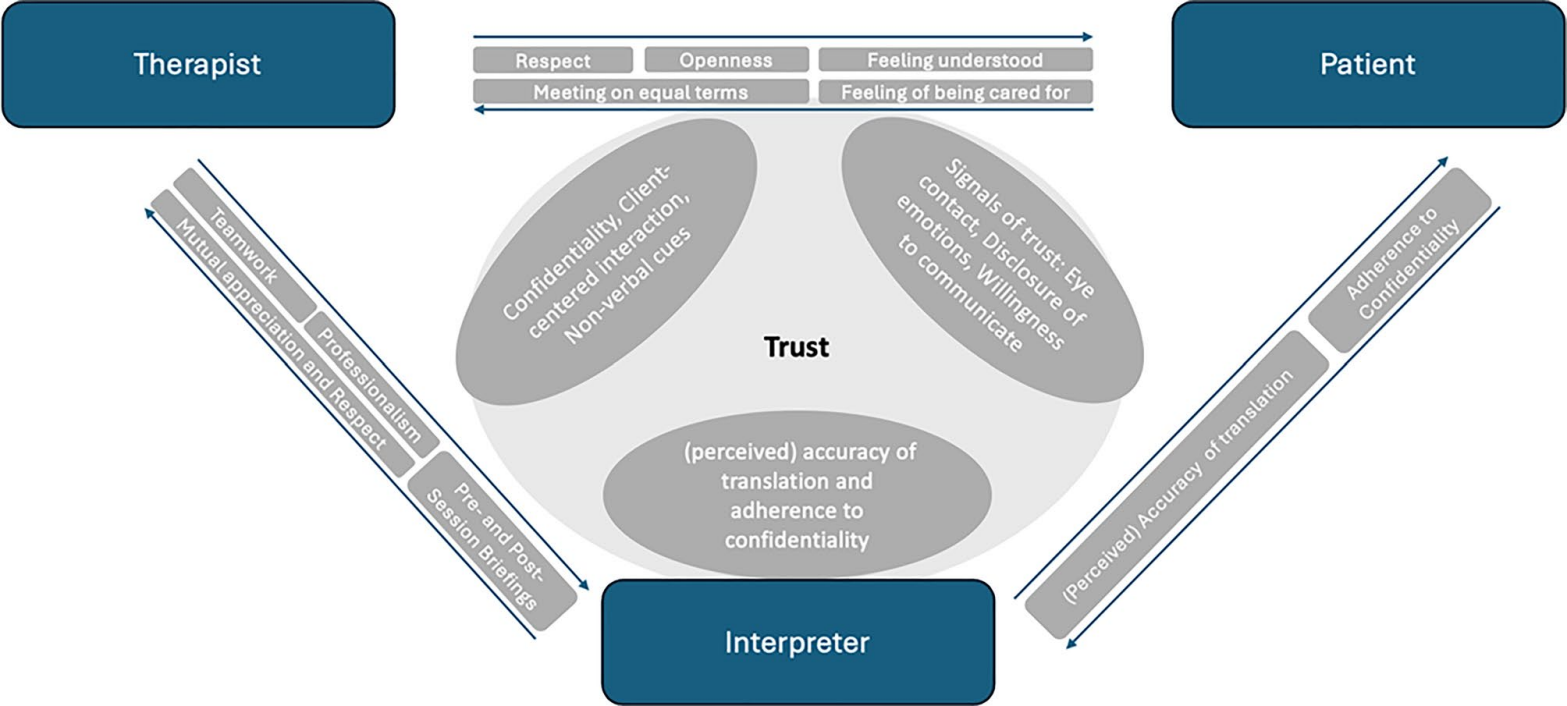
Overview of identified relational qualities and trust-building aspects within the triad

The aim of this study was to identify the relationship qualities that shape relationships within the complex relational structures in interpreter-mediated psychotherapies. To achieve this, the perspectives of all three parties—interpreter, therapist, and patient—were considered. The analysis showed that there are both overlapping global relationship qualities that appear to be equally important for all parties, as well as those that play a more significant role for certain perspectives. Among the central relationship qualities that are important to all parties, trust is particularly highlighted. It is considered a fundamental prerequisite for successful collaboration within the triad and forms the core of all dyadic relationships. The complexity of different relationship qualities regarding the different dyadic relationships that were identified makes it difficult to present them in a clear and structured way. We developed a visual representation of an initial approach to modelling the general tendencies of key relational qualities within the triadic relationship (Fig. 3).

However, it seems that trust is always closely tied to the relationship qualities. It became evident that trust is not only perceived as a general quality but also unfolds different expectations and functions depending on the specific relationship. While this in line with different research studies, where the importance of trust in interpreter-mediated psychotherapy is mentioned [36, 39, 40], this study explored a nuanced understanding of how trust can be built, what challenges may arise and how to identify if there is trust between the participants.

### Indicators of (therapeutic) relationships in IMP

Against the background of the complex relationship structures in interpreter-mediated settings, it was important to identify key indicators of a good relationship within the IMP. Therapists, patients, and interpreters identified several central aspects of a good (therapeutic) relationship within triadic interactions: *trust*,* mutual respect*,* mutual appreciation*,* empathy*,* sympathy*,* openness*,* professionalism*,* clear role understanding and distribution*,* a feeling of comfort and being cared for*,* and encountering each other on equal terms*. These descriptions reveal the expectations that participants in the triadic setting have regarding their respective dyadic relationships. Notably, contrary to various concerns and reservations about interpreter-mediated psychotherapy [25], this study demonstrates that the nature of relationships and the elements that constitute a good relationship in IMP seem not to fundamentally differ from conventional, monolingual psychotherapy. The therapeutic alliance is often described by similar relational qualities as those, which are identified in our study regarding IMP [47]. However, the way these aspects are established, fostered and mediated do change in IMP, as they cannot be conveyed directly to the patient by language itself but must instead be mediated through the interpreter.

In this context, therapists reported that the core conditions of a good relationship do not change, rather, the main difference lies in the slower transmission of information, which extends the time needed to build the therapeutic relationship. However, this can also be advantageous. According to the expert in relationship-building interviewed for our study, the translation process allows the therapist more time to evaluate the patient’s (nonverbal) reactions and reflect on the next therapeutic steps. This additional time seems particularly beneficial for trainees or less experienced therapists in interpreter-mediated psychotherapy. While our data showed a trend where inexperienced or training therapists expressed more doubts and scepticism about using interpreters in psychotherapy, it is important to note that this assumption cannot be confirmed with certainty.

An additional distinctive feature is the integration of more active and expressive nonverbal aspects of communication in order to establish communication, build a bond and convey trust to patients beyond language alone. While in monolingual therapy, language itself may suffice to build trust and relationship, the intentional use of nonverbal cues becomes indispensable in IMP with this regard.

### Trust in patient-therapist relationship

Building trust in the context of interpreter-mediated psychotherapy (IMP) is often a central concern for therapists and is regarded as a significant challenge that can impact the success of psychotherapy. The present study identified that trust is also considered by patients to be an essential prerequisite for opening up to their therapists. Therapists appear to employ two primary strategies to establish trust with patients in IMP. First, providing clear information about confidentiality is a key factor in fostering a trusting therapeutic relationship. This aligns with existing literature, which reports similar findings [30, 38]. It is particularly important to emphasize that confidentiality assurances should not be a one-time measure but rather a recurring practice, whenever needed, to ensure that patients remain continuously aware that everything shared in therapy remains confidential. Here, the role of the interpreter gets important as well. As the interpreter has the influence to facilitate the patient-therapist relationship, assuring and making clear, that patients understand the aspect of confidentiality is a facilitator for trust in the interpreter-patient relationship as well. Secondly, trust is enhanced when therapists adopt a cautious and patient-centered approach, taking the time to acknowledge and address the specific circumstances of their patients before delving into diagnostics or a “classic” treatment procedure. Given research indicates that the therapeutic relationship itself is more crucial to therapy outcomes than specific therapeutic schools or techniques, this approach appears highly relevant [27, 48]. Notably, this strategy was not only reported by therapists but was also explicitly mentioned by patients as a key reason for their trust in their therapists. This is reflected in the way therapists interact with their patients on an equal level, inquire about their families and personal circumstances, or personally welcome them from the waiting room. This was also reflected in the theme of *attentive support*, which encompassed the perceptions of feeling understood, valued and respected. This aligns with current research studies that aim to improve therapist-patient therapeutic engagement. This approach is also reflected in more recent models of needs-oriented psychotherapy, which emphasize the therapist’s attunement to the individual needs, motives, and expectations of the patient as a central element of the therapeutic relationship [49]. Additionally, Stubbe [50] recommends various strategies for fostering a successful patient-therapist relationship and advocates for a patient-centred approach, in which the primary focus is on exploring the patient’s needs, concerns, and hopes in an empathetic manner. Similar to the strategies identified in this study, it is recommended to respond empathetically to patients’ statements and to show appreciation for their attendance and participation [50].

To achieve this, an equally important strategy is the deliberate and active use of non-verbal communication. In accordance with the literature, since spoken language between the patient and therapist is mediated through an interpreter, non-verbal and paraverbal aspects of communication gain significant importance to build a connection with patients across language barriers [51, 52]. Even when the verbal language is not directly understood, patients seem to observe the reactions and non-verbal behaviour of therapists. Therefore, therapists should consciously enhance their use of non-verbal communication, such as facial expressions and gestures, when narrating or listening to the patient’s experiences. This also includes the emotional validation of what has been shared—through facial expressions, tears, or other forms of emotional involvement—which patients perceive as highly trust-enhancing and as an expression of the therapist’s genuine care. Research also highlights the role of therapists nonverbal behaviours in monolingual therapy as it can either facilitate or hinder the therapist-patient interaction [53]. Within the framework of nonverbal strategies, specific therapeutic techniques were also mentioned, such as synchronising the body language of the other person, which in turn is interpreted as an indicator of a good and positive therapeutic relationship [54].

Notably, this study highlights that trust is not solely shaped by the efforts of therapists and interpreters but is also influenced by the personal characteristics of the patients themselves. This underscores the importance of how the (therapeutic) relationship needs to be approached. Given patients’ life histories, particularly their traumatic experiences, this aspect becomes even more significant. Morina et al. [55] found that refugees often exhibit an avoidant attachment style, which can make building trust particularly challenging. Similarly, insights from patient interviews align with previous research, suggesting that individual life experiences play a crucial role in shaping the ability to establish trust, with some patients encountering significant difficulties in this process [30]. Additionally, also cultural embeddedness, such as the diverging (cultural) norms and values were identified to hinder the establishment of trust. In their study with 485 psychotherapists, Mösko and colleagues [8] found that therapists held similar views, with differing (culturally based) values between therapists and patients being most frequently mentioned as a challenge.

In this context, it is essential to examine how to identify if trust is established. A clear indicator of trust appears to be the continuous presence of patients. Furthermore, the study found that patients’ initiative and willingness to communicate are key indicators of a trusting therapeutic relationship. Specifically, when patients openly express their emotions, show vulnerability, cry, or respond to humour, these behaviours can be interpreted as signs of trust. Additionally, eye contact and the body language of the patient seem to be also a sign of trust towards both the interpreter and therapist. These findings are supported also by existing research. While Resera et al. [42] also explored that eye contact and body language of interpreters are a way to build trust with patient, Hanft-Robert et al. [30] found the same aspects also throughout the interviews with therapists. Our finding regarding humour is also supported by recent research, which describes humour as fostering a sense of closeness with the patient and contributing to a relaxed atmosphere [56].

### Relationship towards interpreters

A fundamental prerequisite for the successful transmission and establishment of any kind of relationship in interpreter-mediated settings seems to be the role of the interpreter. The expectations towards interpreters were ambivalent, though predominantly characterised by the need for a professional, close yet simultaneously distant approach, marked by conscientious and accurate translation and adherence to confidentiality. Research suggests that the bond between therapist and interpreter is a key prerequisite for the establishment of the therapist-patient relationship [44]. This study suggests that a positive therapist-interpreter relationship is characterised by professionalism, a sense of teamwork, mutual respect and appreciation. In light of this findings, pre- and post-session meetings become particularly relevant. These meetings can be used to foster mutual understanding of each other’s working methods, clarify roles, and align expectations. Various studies have also highlighted the need for such pre- and post-session meetings, which, when actively encouraged by therapists, seem to be an important part of the process [37]. Additionally, post-session meetings provide a sense of appreciation from therapists towards interpreters. Consistent with the literature and our expert interview with the expert in translational studies [57], interpreters often criticise that their additional skills and experiences are not adequately recognised. During these meetings, not only can therapy-related discussions and evaluations take place—such as clarifying whether any cultural nuances were misinterpreted—but they also serve as an expression of respect for the interpreter’s skills. Offering post-session meetings after particularly challenging therapy sessions is also a sign of attentiveness and shows that therapists care about the well-being of the interpreters [37]. This, in turn, is beneficial for the relationship between the interpreter and the therapist.

While trust was one of the most important indicators for a good (therapeutic) relationship in IMP, this study demonstrated that mistrust seems to be a significant factor that impairs the development of a trusting relationship between the involved participants. Notably, mistrust was primarily directed towards the role and performance of the interpreter. While, in contrast to the findings of Lay et al. [58], our study did not reveal concerns among therapists regarding interpreters’ adherence to confidentiality, therapists’ reservations were mainly related to the transparency and accuracy of translations. However, Grysten et al. [59] reported that therapists experience discomfort when correcting interpreters due to the concern that the feedback might be taken personally.

As mentioned earlier, two strategies were identified amongst therapists to establish trust with their patients. Equally, there are two identified strategies or issues, which seem to build trust between interpreter and patients. While adhering to the confidentiality is a key factor for patients to be able to trust the interpreters, another important factor was the accuracy of translation. Mistrust with regard to these two aspects, were commonly mentioned amongst patient participants. Those patients who possess basic German language skills, may recognize mistranslations and challenge them, as also observed in the study by Lay et al. [56]. These findings are also in line with Delizée and Michaux [43], who found that patients trust in the interpreters gradually builds up through the accuracy of translations and the adherence to confidentiality. Additionally, the (non-verbal) reactions of therapists play a critical role in how patients evaluate the accuracy of translations of interpreters. If a patient shares something tragic or emotional and the therapist responds in a not expected way, it may be perceived as a mistranslation by the interpreter, as the therapist did not react in a desired way. This is particularly important considering that, even if the interpreter translates correctly, such reactions may still be attributed to the interpreter’s competence and translation quality. This exemplifies the complex and intertwined dynamics of the triadic relationship in IMP.

Therefore, pre- and post-session briefings, in which the therapist and interpreter discuss their approach and expectations, become essential in fostering effective collaboration and preventing misunderstandings [41]. These briefings provide an opportunity to align expectations, clarify roles, and ensure a shared understanding of communication strategies, which ultimately enhances trust in the triadic relationship. Unprofessional behaviour and errors by interpreters are frequently attributed to inadequate training of interpreters [60]. Studies have shown that patients tend to develop greater trust and a more stable therapeutic alliance when trained professional interpreters are used [16]. This underscores the importance of training interpreters in mental health settings, as they play a crucial role in shaping the dynamics of interpreter-mediated psychotherapy and influencing the overall therapeutic outcome.

## Limitations

There are some limitations to this study that must be considered when interpreting its findings. First, although the study included participants from various mental health professions and clinics, there is a potential selection bias, as those who chose to participate may have a more favourable view of interpreter-mediated psychotherapy (IMP). This bias may be particularly relevant for therapists, as the recruitment of this group was particularly challenging. While some therapists did express critical perspectives and highlighted difficulties in working with interpreters, this potential bias should be acknowledged when analysing the results. Second, while the sample included diversity in therapy approaches, interpreter languages, and levels of experience, its size remains limited due to the qualitative approach. Additionally, the gender imbalance among the patients represents another limitation, as gender dynamics can play a crucial role regarding trust-building and may therefore limit the perspectives on this topic. The findings may not fully generalize to other regions, healthcare systems, or cultural contexts. Third, as is inherent in qualitative research, the data relies on self-reported accounts, which may be shaped by participants’ personal expectations or social desirability rather than entirely candid reflections. Although several measures were taken to minimise bias, the authors’ cultural and linguistic positions might have shaped the interpretation of the data as well. Future research could complement these findings with observational methods or quantitative analyses to provide further validation.

A key strength of this study lies in its inclusion of all three perspectives within the triadic therapeutic relationship. While most previous research has focused on a single group [30, 36, 37, 61] or at most two [38, 40, 41, 59], this study is one of few to incorporate insights from all three, including the often-overlooked perspective of patients. Given that language barriers among researchers frequently lead to the underrepresentation of patients’ perspectives, this study addresses a critical gap by placing particular emphasis on their experiences. Another notable strength is the multilingual composition of our research team, which allowed for interviews to be conducted in patients’ native languages. Unlike other studies that relied on interpreters during data collection, our approach eliminated the need for mediation, thereby preventing the replication of the very setting which we are investigating. This method provided more direct and unfiltered insights from patients, enhancing the authenticity and validity of the findings.

## Conclusion

This study has highlighted the key relational qualities that contribute to a strong therapeutic alliance within the triadic relationship structure. However, relationship-building in interpreter-mediated therapy presents several challenges that must be carefully considered. While research has explored the characteristics and difficulties of working with interpreters, the core challenge of this triadic setting lies in its very nature. The therapeutic relationship involves three individuals from different backgrounds, and interpreters can vary significantly in their level of training and professionalism. This variability presents not only practical challenges for therapy but also methodological difficulties for research.

Defining the role of interpreters in psychotherapy remains complex and ambiguous. In some cases, their presence is perceived as a barrier to building trust—similar to certain findings in this study. However, other experiences, which were reported in this very same study, suggest the opposite: for some patients, the mere presence of an interpreter fosters a sense of security simply through shared language. The role of interpreters is further complicated by the personal and social dynamics at play. Many interpreters have experienced displacement or migration for reasons similar to those of the patients, making certain topics potentially triggering for them. Additionally, marginalized communities are often tightly connected, meaning interpreters may not be strangers to patients or may be perceived as part of the same social world. This familiarity can sometimes hinder the therapeutic relationship rather than facilitate it. In some instances, patients may even view interpreters from their own cultural background with scepticism. Such perceptions can significantly impact the therapeutic process [60]. Moreover, gender dynamics, such as a perceived mismatch in gender between interpreter and patient may create discomfort or impair openness when discussing sensitive topics, whereas perceived gender alignment may lead, for some, to enhanced trust and sense of comfort. Given the limited data and the individuality of each setting, it may be neither possible nor desirable to formulate rules for gender mismatches and alignments, nevertheless, it is essential to consider these aspects when working with patients and interpreters.

Given this complexity, it is clear that more research is needed to fully understand the role of interpreters and the complex interplay of the relationship dynamics in interpreter-mediated psychotherapy. Their function is highly ambivalent, varying across different contexts and patient experiences. Recognizing this complexity is essential for developing best practices that enhance the therapeutic process. Future research should aim to provide clearer insights into the conditions under which interpreters support or impede therapy and explore strategies to optimize their role in fostering trust and engagement. Particularly, studies with larger sample sizes and quantitative measures, specifically questionnaires to assess the (therapeutic) alliances, the qualities in IMP and triadic trust are necessary to draw more conclusive findings and offer a broader, more generalizable understanding of the dynamics in interpreter-mediated psychotherapy. For this, future research could also draw on findings from psychotherapy with deaf patients, where comparable challenges of communication and trust have been explored. Lastly, while recognizing the complex and challenging nature of this research, it is important to emphasize its necessity: Research on IMP is not merely the product of innovation in psychotherapy but rather a critical effort to overcome language barriers and ensure access to mental health services for individuals who would otherwise be unable to receive them.

## Data Availability

The data sets generated and/or analysed during the current study are not publicly available due to confidentiality considerations.
